# Sentinel factors for mild cognitive impairment in type 2 diabetes mellitus and their interaction mechanisms: a narrative review with network analysis perspective

**DOI:** 10.3389/fendo.2026.1829014

**Published:** 2026-04-29

**Authors:** Qiongqiong Sun, LingYan Zhang, Jianwen Zhao, Shanshan Wang

**Affiliations:** Yangzhou Hospital of Traditional Chinese Medicine (TCM), Yangzhou, China

**Keywords:** cognitive protection, mild cognitive impairment, network analysis, risk factors, sentinel factors, type 2 diabetes mellitus

## Abstract

Type 2 diabetes mellitus (T2DM) is a global chronic metabolic epidemic, and affected patients face a 1.5-fold higher risk of mild cognitive impairment (MCI) — a reversible pre-dementia stage — than non-diabetic populations. Early identification of modifiable sentinel factors is the cornerstone of delaying cognitive decline in T2DM patients, yet traditional statistical methods cannot fully elucidate the synergistic interactions between multi-dimensional risk factors. Network analysis, a graph theory-based statistical approach, enables visualization of complex variable associations and identification of core regulatory nodes in disease networks, providing a novel perspective for MCI research. Based on our team’s clinical data and comprehensive evidence synthesis, this review systematically summarizes the pathophysiological mechanisms linking T2DM and MCI, establishes an operational definition and classification framework of MCI sentinel factors in T2DM patients, elaborates the application value of network analysis in this field, and proposes concrete research paradigms for future investigation. This review aims to provide a theoretical and practical framework for the early screening, targeted intervention, and precise prevention of MCI in T2DM patients, aligning with the scope of clinical diabetes and geriatric endocrinology research.

## 1 Introduction

Type 2 diabetes mellitus (T2DM) is a chronic metabolic disease characterized by insulin resistance and progressive insufficient insulin secretion, which has become a major global public health challenge (1). According to the latest data from the International Diabetes Federation (IDF), the global number of people living with diabetes reached 537 million in 2022, and is projected to rise to 783 million by 2045, with China accounting for more than 25% of the global diabetic population (2). Long-term chronic hyperglycemia in T2DM can cause multiple systemic complications. Among them, cognitive impairment has attracted increasing clinical and research attention, due to its insidious onset, irreversible late-stage progression, and severe adverse clinical outcomes (3).

Mild cognitive impairment (MCI) is defined as an intermediate state between normal cognitive aging and dementia, with core manifestations of mild impairment in memory, attention and executive function, while the basic activities of daily living remain intact (4). A large number of studies have confirmed that T2DM patients have a 1.5-fold higher risk of developing MCI and a 3-fold higher risk of progressing to Alzheimer’s disease (AD) than non-diabetic individuals (5). Our team’s previous cross-sectional study found that the incidence of MCI in 340 T2DM patients was 46.47%, which was consistent with the domestic epidemiological data of about 45%, and the incidence increased significantly with age and prolonged disease duration (6). Once MCI progresses to dementia, there is a lack of effective disease-modifying treatments, which brings a heavy care burden to patients’ families and enormous pressure on the social medical system (7). Therefore, early identification of high-risk individuals and implementation of targeted interventions are critical to delay cognitive decline in T2DM patients.

For this review, we provide a clear, operational definition of sentinel factors for MCI in T2DM patients: early detectable markers that can predict the onset and progression of MCI before the appearance of clinical cognitive symptoms, which must simultaneously meet 4 core criteria: (1) Prospective early warning: abnormal changes can be detected before MCI onset; (2) Clinical monitorability: repeatable, non-invasive/minimally invasive detection via routine clinical means; (3) Robust association: independent statistical correlation with MCI occurrence/progression in T2DM populations verified by at least 2 independent studies; (4) Modifiability: intervention on the factor can alter the risk of MCI. This definition clearly distinguishes sentinel factors from general risk factors, which only require statistical association with the outcome without the above early warning and modifiability attributes (8).

Accurate identification of sentinel factors for MCI in T2DM patients can provide early warning signals before the onset of obvious cognitive symptoms, which is the premise of early intervention. Previous studies have identified a variety of factors associated with MCI in T2DM patients, but most of them focused on the independent effect of single-dimensional factors, and lacked systematic exploration of the complex interactions between multi-dimensional factors (9). Notably, most existing evidence is derived from cross-sectional studies, which can only reveal associations between variables but cannot clarify causal relationships or temporal sequence between factor exposure and MCI onset (10).

Network analysis is an emerging statistical method derived from graph theory, which treats variables as “nodes” and the partial correlation between variables as “edges” to construct a network model that intuitively presents the complex interactions between multiple variables (4). Through centrality analysis, network analysis can accurately identify core regulatory nodes in the network, providing a new perspective for exploring the interaction mechanism of disease risk factors and prioritizing intervention targets (11). In recent years, network analysis has been widely used in psychopathology and chronic disease management, but its application in the study of MCI sentinel factors in T2DM patients is still limited (12).

Based on our team’s previous research results and comprehensive synthesis of global independent evidence, this review systematically collates the pathophysiological mechanisms linking T2DM and MCI, categorizes and summarizes the research progress of MCI sentinel factors in T2DM patients, elaborates the application of network analysis in this field, and puts forward the current challenges and concrete actionable future research prospects. This review provides a comprehensive theoretical framework for the early warning and precise intervention of MCI in T2DM patients, and has good clinical reference value and academic publication adaptability.

## 2 Pathophysiological mechanisms linking T2DM and MCI

**Figure 1:**
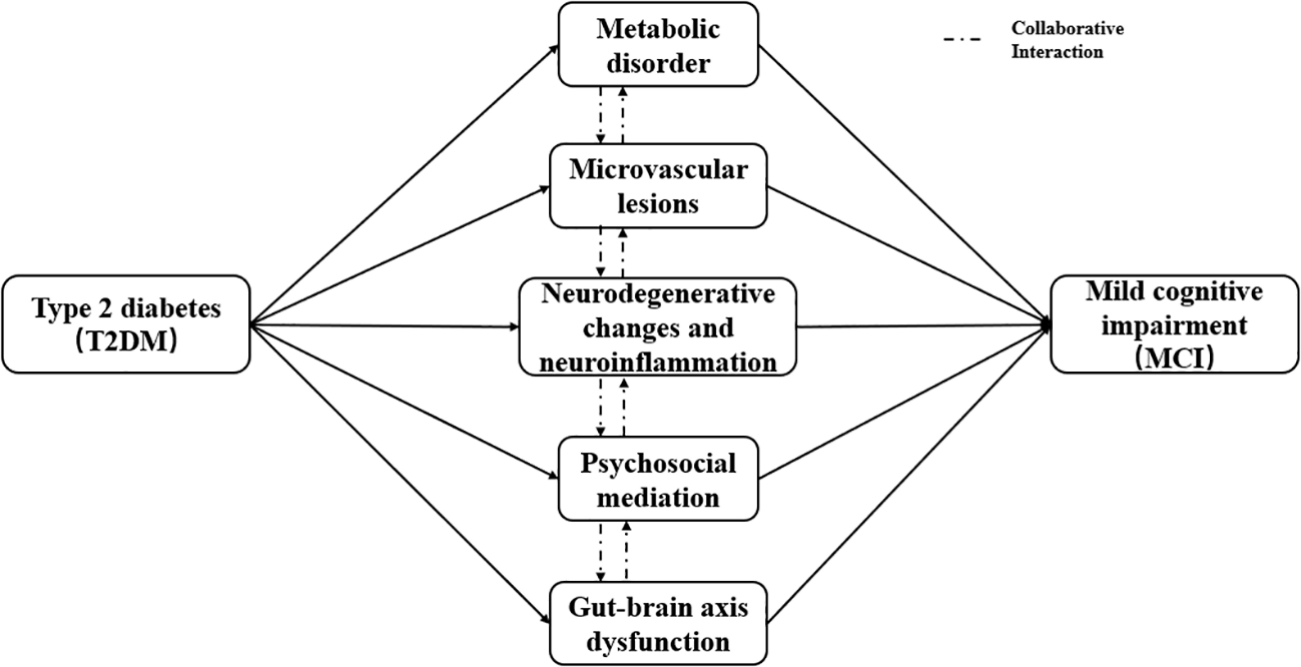
Schematic diagram of pathophysiological mechanisms linking T2DM and MCI.

The occurrence of MCI in T2DM patients is the result of the synergistic effect of multiple intertwined pathological mechanisms, which jointly promote the progression of cognitive impairment (13). The main mechanisms can be divided into five interrelated categories, which have been fully verified in basic and clinical studies (**Figure 1**).

### 2.1 Metabolic disorder-related mechanisms

Chronic hyperglycemia is the core initiating factor of cognitive impairment in T2DM patients. Long-term high glucose can abnormally activate the polyol pathway and hexosamine pathway, leading to the massive production of reactive oxygen species (ROS) and triggering oxidative stress reactions (14). ROS can damage the DNA, proteins and lipids of nerve cells, leading to neuronal dysfunction and apoptosis. In addition, hyperglycemia promotes the production and accumulation of advanced glycation end products (AGEs). AGEs bind to receptors on the surface of nerve cells, further aggravating oxidative stress and inflammatory responses, and destroying the integrity of the nerve cell membrane (15).

Insulin resistance is another core mechanism. The central nervous system also has a complete insulin signaling pathway, which is involved in the regulation of neuronal metabolism, survival and synaptic plasticity. Central insulin resistance in T2DM can lead to dysfunction of this pathway, resulting in neuronal energy metabolism disorder and impaired memory and executive function (16). Meanwhile, insulin resistance promotes the production and deposition of β-amyloid (Aβ) and inhibits its clearance, which is a core pathological feature of AD (17). Recurrent hypoglycemic episodes also have a non-negligible damaging effect on cognitive function. Acute severe hypoglycemia leads to insufficient cerebral energy supply, causing neuronal edema and necrosis, especially in the hippocampus which is closely related to cognitive function (18).

### 2.2 Microvascular lesion mechanisms

Diabetic microvascular disease is a core pathological basis mediating cognitive impairment in T2DM patients. Long-term hyperglycemia directly damages the endothelial cells of cerebral microvessels, leading to thickening of the vascular wall, stenosis of the lumen, and disturbance of cerebral hemodynamics, resulting in chronic cerebral ischemia and hypoxia (19). Cerebral microcirculation disorder inhibits the aerobic metabolism of neurons, reduces ATP production, and blocks the transport of nutrients to nerve cells, leading to decreased synaptic plasticity and impaired neural circuit function.

Notably, diabetic retinopathy (DR), a peripheral microvascular complication of diabetes, shares the same pathological pathway of “hyperglycemia-induced endothelial injury - vascular remodeling - microcirculation disorder” with cerebral microvascular disease. Multiple large-scale prospective cohort studies have confirmed that T2DM patients with DR have a 2.3-fold higher risk of cognitive impairment than those without complications, so DR can be used as a “window” of cerebral microvascular disease to indirectly reflect the risk of cognitive impairment (20). In addition, cerebral microvascular disease directly damages the physiological function of the blood-brain barrier (BBB). Hyperglycemia down-regulates the expression of tight junction proteins in the BBB, leading to increased vascular permeability, which allows inflammatory factors in the blood to enter the brain tissue and hinders the clearance of Aβ (21).

### 2.3 Neurodegenerative change and neuroinflammation mechanisms

T2DM patients have significant neurodegenerative changes in the brain, mainly manifested as neuronal loss, synaptic dysfunction, neurofibrillary tangles and senile plaque formation (17). Hyperglycemia and insulin resistance promote the production and deposition of Aβ, which forms oligomers and exerts neurotoxic effects, leading to neuronal apoptosis. Meanwhile, T2DM promotes the hyperphosphorylation of tau protein, which aggregates to form neurofibrillary tangles, destroys the cytoskeleton of nerve cells, and impairs nerve signal transmission (22).

Chronic neuroinflammation plays a key role in T2DM-related cognitive impairment. Long-term hyperglycemia activates microglia in the brain, which continuously release pro-inflammatory factors such as tumor necrosis factor-α (TNF-α) and interleukin-6 (IL-6). These inflammatory factors directly damage neurons, inhibit the proliferation and differentiation of neural stem cells, and destroy synaptic plasticity, leading to cognitive decline (23). In addition, peripheral inflammation and central inflammation interact with each other, forming a vicious cycle that further aggravates the neuroinflammatory response (24).

### 2.4 Psychosocial factor-mediated mechanisms

Psychosocial factors play an important mediating role in the occurrence of MCI in T2DM patients. Depression is a common psychological problem in T2DM patients, with an incidence of about 20%~30% (25). Depression affects cognitive function through multiple pathways: it causes the disturbance of neurotransmitters such as serotonin and dopamine, leads to over-activation of the hypothalamic-pituitary-adrenal (HPA) axis and increased cortisol secretion which damages the hippocampus, and causes behavioral changes such as lack of exercise and sleep disorders, which indirectly aggravate cognitive impairment (26).

Sleep disturbance is also an important mediating factor. T2DM patients often have sleep problems such as sleep apnea syndrome, frequent nocturnal awakening caused by polyuria or peripheral neuropathy pain. Sleep is a critical period for the clearance of metabolic waste in the brain, and sleep disorders can block the clearance of Aβ (27). In addition, sleep deficiency leads to the disturbance of neurotransmitter secretion and the activation of systemic inflammatory response, which jointly aggravate cognitive function damage. Inadequate social support also increases the risk of MCI, as good social support can protect cognitive function by relieving psychological stress, promoting healthy behaviors and improving medication adherence for glycemic control (28).

### 2.5 Gut-brain axis dysregulation and nutritional metabolic mechanisms

Emerging evidence has confirmed that gut-brain axis dysregulation is a key mechanistic link between T2DM and MCI. T2DM is characterized by gut microbiota dysbiosis, including reduced abundance of beneficial bacteria (e.g., Bifidobacterium, Lactobacillus) and overgrowth of pro-inflammatory pathogenic bacteria (29). Dysbiotic gut microbiota disrupts intestinal barrier integrity, triggers low-grade systemic inflammation, and promotes the translocation of bacterial metabolites and pro-inflammatory factors into the systemic circulation, further aggravating central neuroinflammation and neuronal damage (30). In addition, gut microbiota metabolites such as short-chain fatty acids (SCFAs), tryptophan metabolites, and lipopolysaccharides can directly regulate microglial activation, Aβ clearance, and synaptic plasticity via the vagus nerve or systemic circulation, thereby affecting cognitive function (31).

Nutritional and dietary factors are critical modulators of gut microbiota function and glycemic regulation in T2DM patients. Tropical herbs and their bioactive compounds have been shown to modulate gut microbiota composition, improve intestinal barrier function, enhance insulin sensitivity, and reduce systemic inflammation, which may exert indirect protective effects on cognitive function in T2DM patients (32). Dietary patterns such as the Mediterranean diet and low-glycemic index diet have also been associated with reduced MCI risk in T2DM populations, making dietary and nutritional status a potential modifiable target for early intervention (33).

## 3 Sentinel factors for MCI in T2DM patients

Based on the operational definition and 4 core criteria of sentinel factors and our team’s previous research, the sentinel factors can be divided into three categories: biomarker-based, clinical indicator-based, and psychosocial and behavioral sentinel factors, which are systematically summarized below.

### 3.1 Biomarker-based sentinel factors

Biomarkers are objective laboratory indicators that can reflect the pathophysiological changes in the body, and are the core components of the sentinel factor system. Our team’s previous cross-sectional study identified two core biomarker-based sentinel factors: glycated hemoglobin (HbA1c) ≥7.0% and homocysteine (HCY) ≥15μmol/L, both of which are independent risk factors for MCI in T2DM patients (6).

HbA1c, which reflects the average blood glucose level in the past 2–3 months, is the most widely studied glycemic control biomarker. A 5-year prospective cohort study and multiple meta-analyses have confirmed that HbA1c level is negatively correlated with cognitive function in T2DM patients, and the risk of MCI increases significantly when HbA1c ≥7.0% (34). A 5-year cohort study found that for every 1% increase in HbA1c, the rate of cognitive decline in T2DM patients increases by 15%, which is consistent with our research results that HbA1c ≥7.0% increased the risk of MCI by 2.418 times (OR = 2.418, 95% CI:1.386~4.219) (6). Other core biomarkers in this category all meet the 4 criteria of sentinel factors, with clear clinical detection methods and modifiable intervention approaches (**Table 1**).

**Table 1:** Classification framework and core criteria of sentinel factors for MCI in T2DM patients.

Category of sentinel factors	Core sentinel factors	Evidence level	Modifiability
Biomarker-Based	Glycated Hemoglobin (HbA1c) ≥7.0%	Longitudinal cohort study	Yes (Improvable via hypoglycemic intervention)
	Homocysteine (HCY) ≥15μmol/L	Longitudinal cohort study	Yes (Reducible via folic acid/vitamin B12 supplementation)
	Lipoprotein a (Lp(a)) ≥300mg/dL (≈75nmol/L)	Cross-sectional study	Yes (Modifiable with certain medications)
	Inflammatory factors (TNF-α, IL-6)	Cross-sectional study	Partially modifiable (Anti-inflammatory/lifestyle interventions)
	Abnormal Fasting Plasma Glucose (FPG)/2-hour Postprandial Blood Glucose (2hPG)	Cross-sectional study	Yes (Improvable via hypoglycemic treatment)
Clinical Indicator-Based	Age ≥65 years	Longitudinal neuroimaging cohort study	No (Non-modifiable)
	Education level (Primary school or below)	Cross-sectional study (supported by multiple population cohorts)	No (Lifelong stable characteristic)
	Diabetes duration ≥10 years	Longitudinal cohort study	Partially modifiable (Disease management delays progression)
	Complicated with Diabetic Retinopathy (DR)	Longitudinal cohort study	Yes (Delayable via glycemic control/eye protection interventions)
	History of hypertension	Cross-sectional study	Yes (Controllable via antihypertensive treatment)
	Chronic kidney disease	Cross-sectional study	Yes (Delayable via renal protective treatment)
Psychosocial and Behavioral	Moderate to severe depression	Longitudinal cohort study	Yes (Improvable via psychological intervention/medication)
	Poor sleep quality (PSQI ≥8 points)	Longitudinal cohort study	Yes (Improvable via sleep intervention)
	Regular exercise	Supported by Randomized Controlled Trials (RCTs)	Yes (Developable via behavioral intervention)
	Good social network support	Cross-sectional study (cohort-supported)	Yes (Optimizable via social support construction)
	Healthy dietary patterns (Mediterranean diet/low-GI diet)	Cohort study	Yes (Achievable via dietary adjustment)

### 3.2 Clinical indicator-based sentinel factors

Clinical indicator-based sentinel factors can be easily obtained through medical history inquiry and clinical examination, and are suitable for primary screening of high-risk groups in clinical practice. Our team’s study identified four clinical indicator-based sentinel factors: age ≥65 years, education level of primary school and below, diabetes duration ≥10 years, and combined DR (6).

Age and education level are the most basic demographic sentinel factors. Our study found that age ≥65 years increased the risk of MCI by 2.876 times (OR = 2.876, 95% CI:1.632~5.071), and education level of primary school and below increased the risk by 3.124 times (OR = 3.124, 95% CI:1.789~5.468) (6). These two factors are stable lifelong characteristics, which can be used for initial screening of high-risk groups.

Diabetes duration and DR are core diabetes-related sentinel factors. Our study showed that diabetes duration ≥10 years increased the risk of MCI by 2.543 times (OR = 2.543, 95% CI:1.452~4.451), and combined DR increased the risk by 3.367 times (OR = 3.367, 95% CI:1.892~5.994), which was the strongest risk factor in our study (6). In addition, diabetic peripheral neuropathy, hypertension and chronic kidney disease are also important clinical sentinel factors, which are closely related to the increased risk of MCI in T2DM patients (**Table 1**).

### 3.3 Psychosocial and behavioral sentinel factors

Psychosocial and behavioral factors are modifiable sentinel factors, which not only are associated with the occurrence of MCI, but also provide clear intervention targets for clinical practice. Our team’s study identified four psychosocial and behavioral sentinel factors: moderate to severe depression, poor sleep quality (PSQI ≥8 points), regular exercise, and good social network support, among which the first two are risk factors and the latter two are protective factors (6).

Depressive state is a strong risk factor for MCI in T2DM patients. Our study found that moderate to severe depression increased the risk of MCI by 3.015 times (OR = 3.015, 95% CI:1.698~5.354) (6). Longitudinal studies have confirmed that depressive symptoms precede cognitive decline in T2DM patients, supporting its role as a sentinel factor with early warning value (35). Poor sleep quality is another important psychosocial sentinel factor, and our study showed that PSQI ≥8 points increased the risk of MCI by 2.672 times (OR = 2.672, 95% CI:1.523~4.681) (6). Sleep intervention can effectively improve cognitive function in T2DM patients, which confirms the value of sleep quality as a modifiable sentinel factor (36).

Regular exercise and good social network support are two protective sentinel factors. Our study found that regular exercise reduced the risk of MCI by 54.8% (OR = 0.452, 95% CI:0.261~0.783), and good social network support reduced the risk by 61.3% (OR = 0.387, 95% CI:0.221~0.677) (6). In addition, healthy dietary patterns and nutritional status, as modifiable factors regulating gut microbiota and glycemic control, are emerging protective sentinel factors for MCI in T2DM patients (**Table 1**). These factors are easy to intervene in clinical practice, and are important components of the MCI prevention and control system for T2DM patients.

### 3.4 Heterogeneity of sentinel factors across stratified clinical populations

The early warning value of sentinel factors for MCI has significant heterogeneity in T2DM patients with different demographic characteristics and disease courses. Clarifying the stratified differences of core sentinel factors can provide a more accurate basis for clinical personalized screening and stratified management.

#### 3.4.1 Age-stratified heterogeneity

Significant differences in core sentinel factors were observed between elderly (≥65 years) and young-to-middle-aged (<65 years) T2DM patients. For elderly T2DM patients, non-modifiable demographic factors (age, low education level) and diabetes-related clinical indicators (diabetes duration ≥10 years, DR) are the core sentinel factors, with the highest effect size for MCI risk prediction. This is consistent with the cognitive reserve theory and cumulative hyperglycemia injury mechanism, as long-term hyperglycemia exposure and physiological brain atrophy have become the main drivers of cognitive decline in this population (37, 38).

For young-to-middle-aged T2DM patients, modifiable factors are the core warning indicators, including poor glycemic control (HbA1c ≥7.0%, large glycemic variability), moderate-to-severe depression, poor sleep quality, and unhealthy lifestyle (lack of exercise, unhealthy dietary patterns). These factors have a more significant impact on cognitive decline in this population, and early standardized intervention can achieve greater cognitive protection benefits and delay the onset of cognitive impairment.

#### 3.4.2 Gender-stratified heterogeneity

Gender differences exist in the sentinel factors for MCI in T2DM patients. For female T2DM patients, psychosocial factors (moderate-to-severe depression, poor social support) and nutritional metabolic indicators (homocysteine level) have stronger early warning value for MCI. This may be related to the dramatic fluctuation of estrogen levels after menopause, the higher prevalence of depression, and the higher risk of malnutrition in elderly women.

For male T2DM patients, vascular-related clinical indicators (hypertension, chronic kidney disease, smoking history) and inflammatory biomarkers have a more significant correlation with MCI risk. This is consistent with the higher susceptibility of male patients to vascular endothelial injury and chronic inflammatory response under chronic hyperglycemia exposure.

#### 3.4.3 Diabetes duration-stratified heterogeneity

The core sentinel factors vary significantly with the duration of T2DM. For patients with T2DM duration <10 years, glycemic control-related biomarkers (HbA1c, FPG, 2hPG) and lifestyle behavioral factors are the core sentinel factors. The main pathological change in this stage is reversible metabolic disorder without significant irreversible microvascular injury, so early intervention of glycemic control and lifestyle can effectively reduce the risk of MCI.

For patients with T2DM duration ≥10 years, microvascular complication indicators (DR, chronic kidney disease), neurodegeneration-related biomarkers (HCY, inflammatory factors), and age are the core sentinel factors. Long-term cumulative hyperglycemia has led to irreversible cerebral microvascular injury and neuronal damage in this population, which are the main drivers of cognitive decline, and the intervention focus should be on delaying the progression of complications and neuroprotective treatment.

## 4 Application of network analysis in T2DM-related MCI research

Traditional statistical methods mostly focus on the independent effect of single factors on the outcome, and cannot fully elucidate the synergistic interactions between multi-dimensional sentinel factors. Network analysis, as a graph theory-based statistical method, can intuitively visualize the complex associations between variables through node-edge network models, and accurately identify core regulatory nodes in the disease network through centrality analysis. This provides a new methodological perspective for exploring the interaction mechanism of sentinel factors and prioritizing intervention targets (4).

### 4.1 Basic principles of network analysis

Traditional statistical methods mostly focus on the independent effect of single factors on the outcome, and cannot fully elucidate the synergistic interactions between multi-dimensional sentinel factors. Network analysis, as a graph theory-based statistical method, can intuitively visualize the complex associations between variables through node-edge network models, and accurately identify core regulatory nodes in the disease network through centrality analysis. This provides a new methodological perspective for exploring the interaction mechanism of sentinel factors and prioritizing intervention targets (4).

### 4.2 Research progress of network analysis in this field

**Figure 2:**
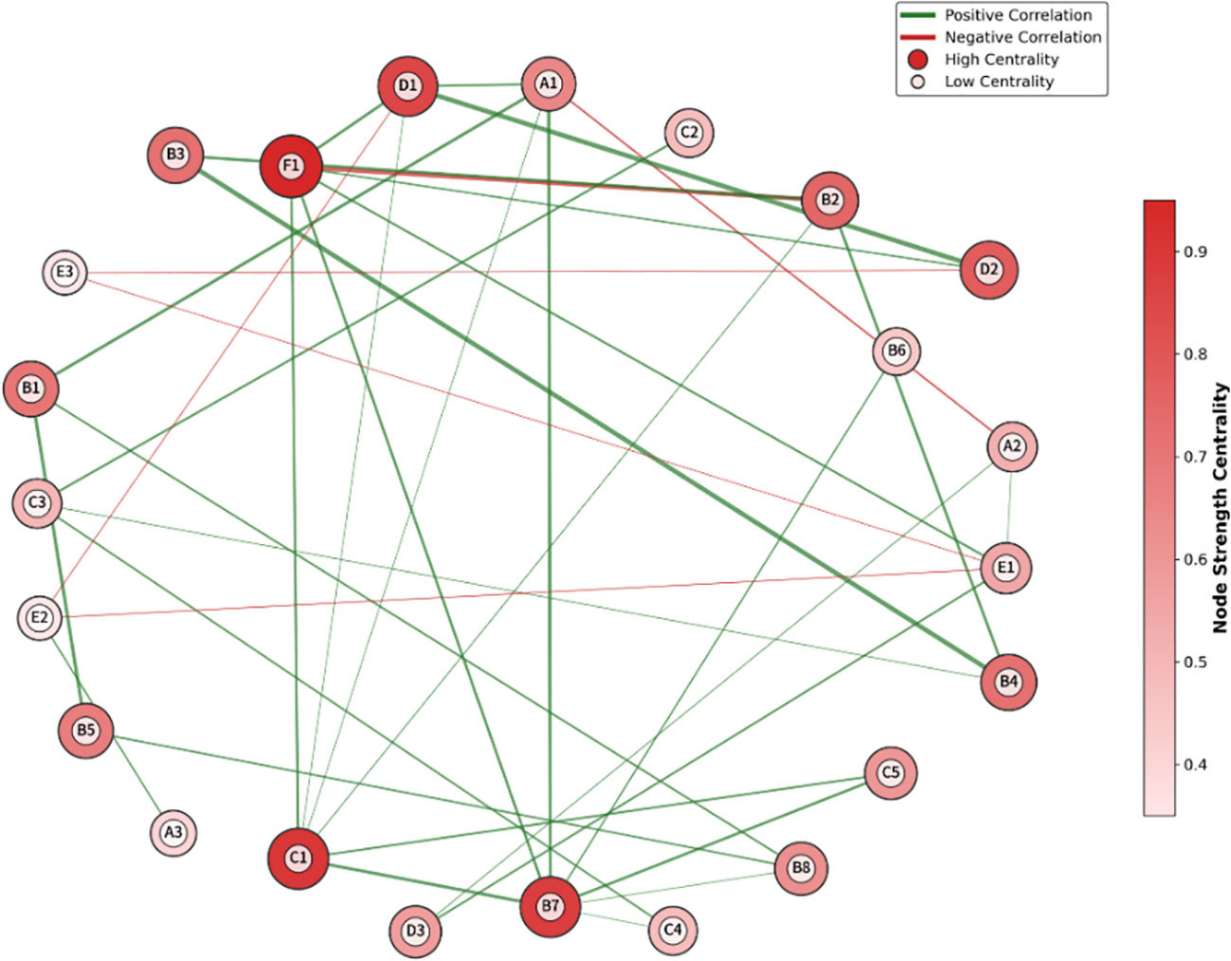
Gaussian graphical model of MCI risk factors in T2DM patients. A1, (Age); A2, (Education Level); A3, (Gender); B1, (Disease Duration); B2, (HbA1c); B3, (FPG); B4, (2hPG); B5, (DR); B6, (Diabetic Neuropathy); B7, (Hypertension); B8, (CKD); C1, (HCY); C2, (Total Cholesterol); C3, (Triglycerides); C4, (LDL-C); C5, (Lp(a)); D1, (Depression); D2, (Sleep Quality); D3, (Social Support); E1, (Regular Exercise); E2, (Smoking); E3, (Alcohol Consumption); F1, (Glycemic Control).

Our team previously constructed a Gaussian Graphical Model (GGM) network of MCI sentinel factors in T2DM patients based on clinical data, which included 22 nodes (sentinel factors) and 38 edges (statistically significant partial correlations between factors) (**Figure 2**). Through centrality analysis, we identified 8 core regulatory nodes with the highest strength centrality, including DR, HbA1c ≥7.0%, moderate-to-severe depression, diabetes duration ≥10 years, HCY ≥15μmol/L, poor sleep quality, age ≥65 years, and low education level. These core nodes have the strongest connectivity with other factors in the network, and are the key targets for priority intervention.

## 5 Current challenges and future perspectives

### 5.1 Clinical translation value of sentinel factors and network analysis

The sentinel factor system established in this review can be used for the whole-process management of MCI in T2DM patients, including initial screening of high-risk groups, precise risk assessment, and targeted intervention. Network analysis can further identify the core regulatory nodes in the risk network, and provide a scientific basis for the formulation of multi-target synergistic intervention strategies. The integration of the two can effectively promote the transformation of research results into clinical practice.

### 5.2 Future research prospects

First, large-scale multi-center prospective cohort studies are needed to further verify the early warning value of sentinel factors, and clarify the optimal detection threshold and early warning time window of each factor. Second, the application of network analysis in this field needs to be further deepened, and dynamic network models should be constructed to explore the temporal changes of sentinel factor interactions during the progression of cognitive impairment. Finally, randomized controlled trials (RCTs) of multi-target synergistic intervention based on core sentinel factors are needed to confirm the cognitive protective effect of targeted intervention.

## 6 Conclusion

MCI in T2DM patients is the result of the synergistic effect of multiple factors, including metabolic disorders, microvascular lesions, neurodegeneration, neuroinflammation, gut-brain axis dysregulation and psychosocial factors (13). The sentinel factors for MCI in T2DM patients cover biomarker-based, clinical indicator-based, and psychosocial and behavioral factors, among which age ≥65 years, low education level, diabetes duration ≥10 years, DR, HbA1c ≥7.0%, HCY ≥15μmol/L, moderate to severe depression, poor sleep quality are independent risk factors, while regular exercise, good social network support and healthy dietary patterns are protective factors. These sentinel factors can provide early warning signals for the occurrence of MCI, and most of them are modifiable, providing clear targets for clinical intervention.

Network analysis can reveal the complex interactions between multiple risk factors, identify core regulatory nodes, and break through the limitations of traditional statistical methods (39). The integration of sentinel factor identification and network analysis can not only verify the core position of sentinel factors in the risk network, but also reveal the interaction mechanism between factors, providing a scientific basis for the formulation of multi-target synergistic intervention strategies (40).

Despite the current challenges, with the deepening of research, the sentinel factor system will be more comprehensive, and the application of network analysis will be more in-depth (41). The early screening and precise intervention based on sentinel factors and network analysis will effectively delay the cognitive decline of T2DM patients, reduce the incidence of dementia, and improve the quality of life of patients.
